# Exploring the Effects of Antisocial Personality Traits on Brain Potentials during Face Processing

**DOI:** 10.1371/journal.pone.0050283

**Published:** 2012-11-21

**Authors:** Daniela M. Pfabigan, Johanna Alexopoulos, Uta Sailer

**Affiliations:** 1 Social, Cognitive and Affective Neuroscience Unit, Faculty of Psychology, University of Vienna, Vienna, Austria; 2 Department of Psychoanalysis and Psychotherapy, Medical University of Vienna, Vienna, Austria; 3 Department of Psychology, University of Gothenburg, Gothenburg, Sweden; Ecole Normale Supérieure, France

## Abstract

Antisocial individuals are characterized to display self-determined and inconsiderate behavior during social interaction. Furthermore, recognition deficits regarding fearful facial expressions have been observed in antisocial populations. These observations give rise to the question whether or not antisocial behavioral tendencies are associated with deficits in basic processing of social cues. The present study investigated early visual stimulus processing of social stimuli in a group of healthy female individuals with antisocial behavioral tendencies compared to individuals without these tendencies while measuring event-related potentials (P1, N170). To this end, happy and angry faces served as feedback stimuli which were embedded in a gambling task. Results showed processing differences as early as 88–120 ms after feedback onset. Participants low on antisocial traits displayed larger P1 amplitudes than participants high on antisocial traits. No group differences emerged for N170 amplitudes. Attention allocation processes, individual arousal levels as well as face processing are discussed as possible causes of the observed group differences in P1 amplitudes. In summary, the current data suggest that sensory processing of facial stimuli is functionally intact but less ready to respond in healthy individuals with antisocial tendencies.

## Introduction

Antisocial behavior is described as individual behavior lacking consideration for others, no matter whether intentional or through negligence [1]. Clinical manifestations of antisocial behavior are subsumed under the concept of antisocial personality disorder of the DSM-IV classification [2], and the corresponding diagnosis of dissocial personality disorder of the ICD-10 [3]. Both diagnostic manuals agree on disorder characteristics such as lack of respect for social norms, irresponsibility, reckless and irritable aggressive behavior, and lack of remorse or guilt [4]. In favor of a dimensional theoretical account of personality, non-clinical manifestations of antisocial personality disorder characteristics can also be observed in healthy and non-criminal community samples [5]. Recent theoretical accounts of antisocial behavior stress biological, developmental, and social risk factors [6], [7], [8] for developing antisocial personality disorder.

These behavioral peculiarities of antisocials in social situations give rise to the question whether basic processing of social cues is impaired in these individuals. In every-day life, human faces and facial expressions are regarded as valuable social cues [9], because they embody crucial information useful in social exchange situations. Efficient face analysis can be linked to evolutionary aspects of perception, interaction, and communication in social life [10]. In line with this assumption, Marsh and Blair [11] summarized that antisocial populations repeatedly show deficits in recognizing emotional displays in faces, in particular fearful expressions, assessed in behavioral or neuroimaging settings.

Consequently, the question arises whether impaired recognition of facial expressions in antisocials may be due to deficits in basic sensory functions such as visual processing or deficits in cognitive functions such as attention. However, no study has addressed this particular research question in healthy individuals with antisocial tendencies so far. Although attention deficits have been reported in individuals suffering from antisocial personality disorder [12], one has to keep in mind that these individuals might have also suffered from psychopathy. Psychopathy can be regarded as personality construct sharing some conceptual overlap with antisocial personality disorder [13], [14]. A comorbidity of around 30% has been reported for psychopathy and antisocial personality disorder [13], [14]. However, psychopathy focuses on antisocial personality traits mainly reflected in affective-interpersonal deficits whereas antisocial personality disorder emphasizes observable antisocial behavioral tendencies. Thus, the comparability between these two concepts is somewhat limited. Antisocial personality disorder is most likely associated with impulsive-aggressive tendencies of secondary psychopathy. Secondary psychopathy refers to the facet of psychopathy characterized by increased impulsivity and a socially deviant life style [15]. Nevertheless, antisocial traits are also associated with deficits in emotional reactivity and as such reflected in overall diminished skin conductance variability [16], [17] or in recognition deficits of fearful facial expressions [11]. Furthermore, inadequate sensitivity to emotional stimuli and stress reactivity has been observed in antisocial-impulsive aspects of psychopathy [18]. Recently, Verona, Sprague, and Sadeh [19] conducted a direct comparison between psychopathic individuals and individuals suffering from antisocial personality disorder. The authors found psychopathy to be associated with reduced neural processing of negative emotional stimuli, whereas antisocial individuals were associated with prioritized processing of these negative stimuli under inhibitory control requirements.

To determine possible impaired stimulus processing stages, empirical evidence of the precise temporal occurrence of these deficits is necessary. Electrophysiological measures are a useful tool to investigate the time course of face processing. The present study focused on two event-related potentials (ERPs) that have been linked to face processing; the P1 and the N170, respectively. Furthermore, the P300 component was of interest because of its assumed role in attentional processes [20].

The P1 is a positive-going ERP which can be found at parieto-occipital and occipital electrode sites with onset latencies between 60 and 80 ms and peak latencies between 100 and 130 ms after visual presentation [21]. The P1 indexes an early stage of visual processing. Physical stimulus characteristics such as luminance or contrast (i.e., low-level visual features) are reflected in the P1 amplitudes [21]. Indeed, neuronal generators of the P1 component were found within lateral extrastriate areas [22], [23]. However, apart from low-level visual processing, P1 amplitude is also modulated by top-town attentional processes. Its amplitude is reported to be enhanced for attended compared to unattended stimuli, which holds true in particular in paradigms investigating spatial attention [24], [25]. Moreover, P1 has also been linked to face categorization processes [26], [27]. For example, negative emotional faces have been observed to evoke larger P1 components than positive emotional faces [28], [29].

The P1 is usually followed by the N170, an ERP component that has also been linked to early face processing stages [30], [31]. The N170 is a negative-going deflection of the ERP peaking about 130 to 190 ms after stimulus presentation at occipito-temporal electrode sites. Neuronal generators of the N170 are assumed to lie within ventral visual areas (i.e., fusiform gyrus) [30], [32], [33] or within lateral temporal regions [34]. The N170 is reported to be enhanced after facial compared to non-facial stimuli [30], [31]. Eimer [33] related the N170 to structural encoding of facial features, thus responding to faces on a categorical rather than on an individual level [35]. To be more specific, the N170 is sensitive to the presentation of faces in general, but it does not incorporate information about whether a particular face is familiar or not. It remains a question of debate whether the N170 is also sensitive to emotional facial expressions. For example, Batty and Taylor [36] reported enhanced N170 amplitudes after fearful compared to neutral faces as well as shorter N170 peak latencies for positive compared to negative emotional expressions. In contrast, Eimer and Holmes [37] concluded that the N170 amplitude was not at all sensitive to different facial emotional expressions.

The P300 (also P3 or classical P3b) is an ERP deflection at posterior electrode locations, peaking around 300–600 ms after stimulus presentation [38], [39]. P300 amplitude variation depends on factors such as categorical stimulus probability [39], stimulus quality, attention [40], as well as task complexity, resource allocation [41] and arousal states [42]. Findings concerning P300 amplitude variation in antisocials and psychopaths are inconsistent; both amplitude enhancement [43] as well as decrease [44] have been reported.

The present study aimed to investigate early processing stages of facial stimuli in individuals high and low on antisocial traits from a sub-clinical sample in a gambling task context. We chose to administer a gambling task instead of a classical passive viewing task to enhance the salience of the presented faces which served as feedback stimuli. Initially, this task was administered to investigate expected and unexpected feedback outcomes [45]. Behavioral task measures such as reaction times and button choice behavior were assumed to reflect task engagement. The presented feedback faces always incorporated information regarding correctness of a prior response. Furthermore, we chose happy and angry faces representing feedback since the distance between two facial emotional expressions is reported to be maximal from anger to happiness [46]. Notably, no emotion recognition deficits have been reported in antisocial populations concerning anger and happiness displays [11]. Thus, we assumed that participants could easily differentiate between positive and negative feedback.

In accordance with possible attention deficits in antisocial personality disorder [12], we explored whether any deficits in early sensory analysis were observable in healthy individuals scoring high on an antisocial trait measure. If so, we expected the high-trait group to display diminished P1 and N170 components compared to the low-trait group. Regarding stimulus valence, i.e., facial expression, we expected larger P1 amplitudes after negative than positive feedback faces [26], [27], but no modulation of N170 amplitudes [37]. Since Marsh and Blair [11] reported no emotion recognition deficits for anger and happiness in antisocial populations, we did not expect any interaction effects between feedback valence and antisocial traits. Additionally, we explored behavioral task outcomes such as reaction times and button choice behavior.

## Results

### Behavioral Results

The analysis of response times revealed a significant main effect of *cue* (*F*(2,42) = 26.70, *p* = 0.001, *η_p_^2^* = 0.56). Bonferroni-corrected multiple comparisons revealed significantly slower reaction times in response to the 0% cue than to the 75% and 100% cues (all *p*’s <0.001). In contrast, all participants responded with comparable reaction times to the 75% and 100% cues (*p* = 0.453). No *group* effect was found (*F*(1,21) = 0.10, *p* = 0.754), no interaction effect emerged either (*F*(2,42) = 1.15, *p* = 0.328). Comparably, no group difference emerged concerning button choice behavior (*t*(17.65) = 0.42, p = 0.682). Both groups achieved a comparable number of correct responses at the end of the experiment (on average 495+/−21.65).

### P1 Amplitudes

**Figure 1:**
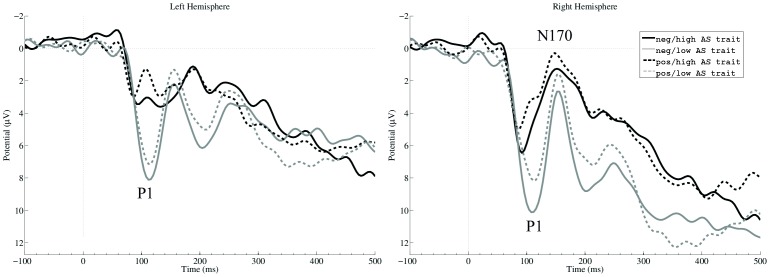
Grand average waveforms. Grand averages of the feedback presentation of low antisocial trait (low AS trait; grey) and high antisocial trait (high AS trait; black) individuals depicted at left (averaged mean amplitudes of L23 and L24) and right (averaged mean amplitudes of R26 and R27) electrode locations. The dotted line at time 0 indicates stimulus onset, negative is drawn upwards per convention.

Grand mean amplitudes of P1 and N170 mean amplitudes after facial feedback presentation are displayed in Figure 1.

**Figure 2:**
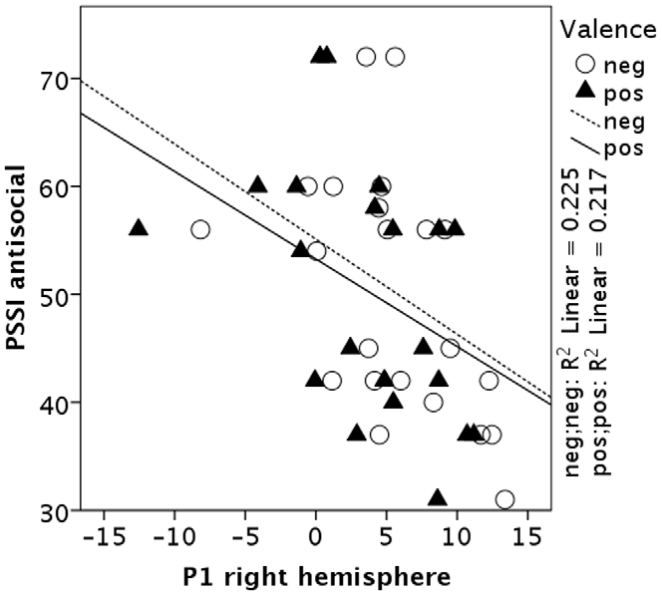
Scatter plot of AS-subscale scores and P1 amplitudes at right-hemispherical electrode locations for happy (triangles) and angry (circles) facial feedback stimuli.

Regarding P1 amplitudes, main effects for the factors *group* (*F*(1,21) = 5.70, *p* = 0.026, *η_p_^2^* = 0.21), *electrode location* (*F*(2,42) = 4.92, *p* = 0.012, *η_p_^2^* = 0.19), and *valence* (*F*(1,21) = 18.08, *p*<0.001, *η_p_^2^* = 0.46) emerged. The low-trait participants showed generally larger P1 amplitudes than high-trait ones, independent of facial stimulus' valence or electrode location. Effects of *electrode location* and *valence* were subsumed under a significant interaction (*F*(1,21) = 33.19, *p*<0.001, *η_p_^2^* = 0.61). Post-hoc tests revealed significantly larger P1 amplitudes after negative compared to positive facial expressions at all electrode sites (all *p*’s<0.001). Furthermore, significant negative correlations emerged between the AS-scale and P1 amplitudes following negative (*r* = −0.47, p = 0.022) and positive (*r* = −0.47, p = 0.025) facial presentation on the right hemisphere. Higher scores on the AS-scale were associated with smaller P1 amplitudes after face presentation for both emotional displays. Correlations did not reach significance at electrodes on the left hemisphere and at Oz (all *p*’s>0.173). Correlations between AS-scale scores and P1 amplitudes at right electrode locations are depicted in Figure 2.

Regarding P1 latency a main effect of *valence* (*F*(1,21) = 5.79, *p* = 0.025, *η_p_^2^* = 0.22) occurred, indicating that P1 amplitude peaked earlier after happy (*M* = 99 ms, *SE* = 3.52) than angry (*M* = 102 ms, *SE* = 3.59) facial stimuli. Neither the factors *electrode location* (*p* = 0.081) and *group* (*p* = 0.596), nor any interaction effects reached significance (all *p*'s>0.456).

### N170 Amplitudes

Regarding N170 amplitudes, a main effect of *valence* (*F*(1,21) = 6.32, *p* = 0.019, *η_p_^2^* = 0.23) emerged. N170 amplitudes were more pronounced after positive compared to negative facial expressions in all participants. Neither the factors *electrode location* (*p*>0.616) and *group* (*p*>0.246), nor any interaction effects (*p*>0.171) reached significance. Correlational analysis revealed also non-significant correlations for N170 amplitudes following negative (all *p*’s>0.140) and positive (all *p*’s>0.260) facial expressions and AS-scores on both hemispheres. Mean amplitudes for P1 and N170 amplitudes are depicted in Table 1.

**Table 1:** Condition-wise (NEG – angry faces; POS – happy faces) averaged mean P1 and N170 amplitudes and standard deviation (SD) for face presentation on the right and left hemisphere and at Oz for participants high and low on antisocial traits (AS traits).

	P1 mean amplitudes											
	NEG-Right		NEG-Left		POS-Right		POS-Left		NEG-Oz		POS-Oz	
	*Mean*	*SD*	*Mean*	*SD*	*Mean*	*SD*	*Mean*	*SD*	*Mean*	*SD*	*Mean*	*SD*
**low AS traits**	9.14	5.84	6.45	5.90	7.34	5.45	5.75	6.06	7.40	8.34	5.01	8.50
**high AS traits**	2.98	4.76	1.84	3.98	1.32	6.32	0.74	5.24	0.12	7.44	−2.50	9.47
	**P1 latencies**											
	**NEG-Right**		**NEG-Left**		**POS-Right**		**POS-Left**		**NEG-Oz**		**POS-Oz**	
	***Mean***	***SD***	***Mean***	***SD***	***Mean***	***SD***	***Mean***	***SD***	***Mean***	***SD***	***Mean***	***SD***
**low AS traits**	204	14.35	210	23.70	199	13.10	210	21.40	200	24.03	196	24.42
**high AS traits**	201	14.97	203	22.70	199	18.00	201	24.10	197	17.26	195	21.25
	**N170 mean amplitudes**											
	**NEG-Right**		**NEG-Left**		**POS-Right**		**POS-Left**		***xxx***		***xxx***	
	***Mean***	***SD***	***Mean***	***SD***	***Mean***	***SD***	***Mean***	***SD***				
**low AS traits**	2.85	5.37	1.43	6.83	2.04	5.71	0.72	6.59				
**high AS traits**	−1.13	5.26	−0.16	5.23	−1.31	5.71	−0.44	4.54				

### P300 Amplitudes

No significant main effects for *group* (*p*>0.888) or *valence* (*p*>0.829) or an interaction effect (*p*>0.102) emerged for P300 amplitudes.

## Discussion

The aim of the present study was to investigate early sensory processing stages of facial stimuli in individuals scoring low or high on an antisocial trait measure in a gambling context. The main finding of our study is that processing differences between low-trait and high-trait participants can be observed within the first 100 ms after stimulus presentation. The low-trait group displayed significantly larger P1 amplitudes than the high-trait group after both happy and angry facial feedback presentation. No processing differences emerged between the two groups when comparing the later and more complex stages of facial stimulus processing indexed by the N170 component.

At first, it seems reasonable to assume differences in low-level visual processing to be accountable for the observed group differences. However, both groups were presented with the same stimuli, and both groups were able to successfully differentiate negatively-valenced from positively-valenced facial expression, as indexed by the main effect of valence. Thus, low-level visual processing cannot explain the observed group differences. On the contrary, attention-related processes and face recognition processes might account for those differences in the P1 time range.

### Attention-related Theoretical Account

To start with the impact of attention on early sensory processing, Luck and Ford [25] reported an association between larger P1 amplitudes and higher attentional resources for stimulus processing. This assumption implies that high-trait participants attended less to facial stimuli than low-trait participants. Notably, on the one hand this can be interpreted as attention deficit in the high-trait group because fewer attentional resources were directed to the feedback stimuli. On the other hand, one might argue that the high-trait group processed the facial stimuli more efficiently than the low-trait group. Supporting the first notion, reduced activation in the right fusiform gyrus (BA 19) and the left lingual gyrus (BA 18) was found during the presentation of happy and fearful faces in a small forensic sample of male psychopaths in comparison to healthy controls [47]. The authors claimed a hypo-responsiveness of these cortical areas during the processing of facial stimuli. Both cortical areas are assumed to be involved in visual stimulus processing. However, these results cannot account for the missing group effect for N170 amplitudes in the present study. Moreover, this hypo-responsiveness might not be reflected in overt behavior, as pointed out by the authors [47]. Along these lines, no group differences were observed in the current study prior to the feedback presentation for reaction times and button choice behavior. Furthermore, mean P1 latency was comparable in both groups. Thus, it remains a question of debate whether decreased P1 amplitudes and hypo-responsiveness in extrastriate areas are reflecting reduced attentional capacities or more efficient stimulus processing. A recent study investigated passive viewing of unpleasant and neutral pictures in non-institutionalized individuals scoring high on a psychopathy screening who had experience with the justice system [48]. The authors observed decreased N1 amplitude modulation, an ERP also sensitive to early attentional orienting [24] in individuals scoring high on the impulsive-antisocial dimension of psychopathy and discussed diminished attention allocation in these individuals, thereby corroborating the present results. Furthermore, Figure 2 in their study [48] displays potential general amplitude differences in the P1 range, although no group-specific plot was provided.

Additionally to attention allocation processes, Luck and Ford [25] claimed that high internal arousal would result in heightened P1 amplitudes. Therefore, another possible interpretation of our results is that the high-trait group might have in general a lower level of internal arousal than the low-trait group. Unfortunately, we did not measure any psychophysiological arousal measures such as skin conductance level or heart rate variability. However, we assessed mean P300 amplitude after feedback onset as electrophysiological arousal correlate [40], but again did not find any group differences during feedback presentation. Thus, no inferences can be drawn concerning internal arousal levels of the current participants. Deficient P300 amplitude variation as summarized by Gao and Raine [49] might only be observable in clinical or sub-clinical populations, but not in a healthy student sample.

### Face-processing Account

P1 amplitude is particularly associated with face categorization processes [26], [27], [34], [50], [51]. Positive and negative information is reported to be differentiated within the P1 time range [29]. Indeed, negatively-valenced complex images are associated with larger P1 amplitudes than positively-valenced or neutral complex images [28], [29]. Both studies presented frequent neutral and infrequent emotional images. ERP variation for negatively-valenced images was found for the P1 time range [29], as well as for later processing stages [28]. The present data, however, do not allow the investigation of later processing stages since feedback stimuli were only presented for 700 ms. Nevertheless, our observation of larger P1 amplitudes after angry compared to happy facial expressions is in line with these results. To explain P1 modulation in response to face categorization processes, it has been suggested that motivationally salient stimuli might automatically attract attentional resources to optimize sensory processing [52]. Actually, Keil’s suggestion [52] was based on Eimer’s postulation [53] that the P1 amplitude reflects a sensory gating mechanism. This sensory gating mechanism is assumed to modulate sensory-perceptual stimulus processing via visuo-spatial attention. According to this view, attention leads to a more rapid or more thorough analysis of stimuli at attended locations. This preferential perceptual processing is manifested in enhanced P1 amplitudes [53]. Referring to the present data, low-trait and high-trait participants were both able to successfully differentiate the valence of facial expressions since no interaction effects emerged. Furthermore, since negatively-valenced stimuli elicited enhanced P1 amplitudes than positively-valenced stimuli, one can assume that they attracted more attentional resources, i.e. were more salient to all participants.

Contrary to our group hypothesis, no differences emerged between low-trait and high-trait participants for the N170 component. Thus, we can assume that both groups were able to categorize the presented stimuli as faces in a comparable way. The observation of group differences for P1, but not for N170 amplitudes, points again in the direction of reduced initial attention allocation in the high-trait group.

### Limitations

As with any empirical investigation, however, the present study has limitations which have to be considered when interpreting the results. Apart from the rather small sample size, which renders the present data as preliminary, we have to address another possible limitation. Only female participants were recruited for the present study. It is a well-known fact that prevalence rates for antisocial personality disorder are higher in men than women [54]. Consequently, research on antisociality and related concepts has focused on male participants. However, assumptions obtained in male antisocials might not be transferable to female antisocials and vice versa. Shirtcliff and colleagues [55] go to such lengths as to argue that the neurobiology of antisocial behavior might be fundamentally different in the two sexes. Therefore, our results add to the limited literature regarding antisocial personality traits in healthy women. Antisociality in women is a prevalent problem in the familial and social context alike. However, further research is needed which directly compares antisociality in women and men.

Additionally, future research should address the question whether the P1 amplitude differences at hand depict a face-specific phenomenon or whether they may be generalizable to other stimulus categories. To this end, upside-down faces as well as non-facial stimuli applied as feedback stimuli should be investigated in future studies.

### Conclusion

To summarize, women with high scores on an antisocial trait measure showed diminished P1 amplitudes compared to women with low scores after happy and angry facial feedback presentation indicating reward or non-reward. We suggest that these group differences can be explained by reduced early attention allocation processes in participants high on antisocial traits. Sensory processing of facial stimuli is functionally intact but less ready to respond in these individuals. Interestingly, no behavioral differences arose in response to this observation. This might be explainable by the subclinical nature of the present sample. In general, we assume that high-trait participants usually allocate less attentional resources to external visual stimulation in comparison to low-trait ones when emotional faces are presented.

## Materials and Methods

### Ethics Statement

The present study was conducted in accordance with the Declaration of Helsinki (revised 1983) and local guidelines of the Faculty of Psychology, University of Vienna. According to the Austrian Universities Act 2002 (UG2002) which held at the time the study was carried out, only medical universities were required to appoint ethics committees for clinical tests, application of medical methods, and applied medical research. Therefore, no ethical approval was required for the present study. Nevertheless, it was ethically approved by the head of the former Brain Research Laboratory of the Faculty of Psychology, University of Vienna, to guarantee high international ethical standards. Written informed consent was given by all participants who could withdraw at any time during the experiment without further consequences.

### Participants and Measures

Initially, 28 female students participated in the study. Two participants had to be excluded from further data analysis due to data acquisition artifacts. The remaining 26 participants were aged between 19 and 32 years (mean age 23.4±3.41). All participants were right-handed, as assessed with the Edinburgh Handedness Inventory [56], had normal or corrected-to-normal vision, and reported no psychiatric history. Each participant received 15 Euros bonus for participation at the end of the experiment.

Prior to EEG data collection, participants completed a personality questionnaire, the PSSI (Persönlichkeits-Stil- und Störungsinventar, PSSI; [57]). The PSSI is a self-assessment tool consisting of 14 sub-scales referring to non-pathological personality constructs implemented in the DSM-IV and ICD-10 diagnostic schemes. The sub-scale *self-determined personality and antisocial personality disorder* (AS-antisociality-scale) was used to differentiate between participants high and low on antisocial traits. The AS scale consists of 10 items characterizing people with self-determined and inconsiderate behavior while achieving individual goals, thereby acting self-confidently, humiliating, and offending in their interaction with others. The items had to be rated on a four-point-scale ranging from ‘statement not applying’ to ‘statement completely applying’. The reliability (Cronbach’s α = .86 - AS-scale) and validity [58] of the PSSI are reported to be satisfactory. Individual raw scores were transformed into the corresponding gender-specific T-values (*M* = 50, *SD* = 10) reported in the PSSI manual for all participants.

The average T-score of the remaining 26 participants was 49.70±10.65, ranging from 31 to 72. Participants were divided into two groups based on a median split. However, three participants were excluded from further analyses because their T-values lay too close to the median. Thus, the final sample consisted of 23 participants. Twelve participants were subjected to the low-trait group (mean T = 40.2±4.02), and eleven participants were subjected to the high-trait group (mean T = 60.0±6.26). T-values of both groups differed significantly from each other (independent samples t-test: *t*(21) = 9.12, *p*<0.001), indicating that the group assignment was successful.

### Task

**Figure 3:**
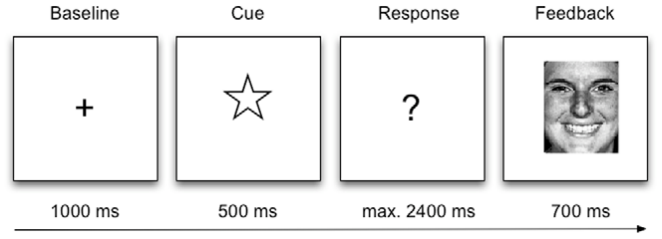
Time line of the gambling task. One of three visual cues (circle, triangle, star) was presented for 500 ms; subsequently, participants had to decide which of two buttons to press considering previously learned cue-response contingencies. After a delay of 400 ms, feedback was presented for 700 ms. Happy faces indicated positive and angry faces indicated negative feedback.

Stimulus presentation (Pentium IV, 3.00 GHz; 19-inch cathode ray tube monitor, Sony GDM-F520; 75 Hz refresh rate) and EEG data collection were synchronized using E-Prime software (Psychology Software Tools, Inc., Pittsburgh, PA). Participants played a gambling task where they were provided with positive and negative feedback stimuli consisting of emotional faces [59], 4×5 cm in size. The paradigm was identical to that described in [45]. Two female and two male faces displaying the emotions happiness and anger were used to indicate positive (happy faces) and negative (angry faces) feedback. Gender and valence of the feedback stimuli were approximately equally distributed during the experiment. Feedback valence was not associated with face identity. Participants were told that the number of positive feedback stimuli (i.e., the number of happy faces) accumulated over the whole experiment, and this number was finally transferred into a monetary bonus. Thus, positive feedback corresponded to reward, negative feedback to non-reward. After a training session of 48 trials where participants learned specific cue-response contingencies, the experimental session started. Participants were told to search for complex cue-response-sequences in a total of 900 trials. After the central presentation of a black fixation cross on a gray screen for 1000 ms, participants were presented with one out of three possible visual cues for 500 ms, namely geometrical line drawings of a circle, a triangle, and a star; 10.5×10.5 cm in size [60]. These three cues depicted reward probabilities of 100, 75 or 0 percent in combination with subsequent button presses which had to be learned in the training session. After the presentation of one of the three cues, a question mark prompted participants to choose one of two buttons on a response pad (index and middle fingers of the right hand) which was placed in front of them. After a response had been made or 2000 ms had elapsed, a delay of 400 ms took place to minimize interference of movement-related brain activity. Subsequently, facial feedback was provided for 700 ms (see Figure 3). However, reward probabilities changed after the training from 100 to 75 and from 0 to 25 percent, respectively; thus participants encountered expected and unexpected feedback. After every 150 trials, participants were provided with overall performance feedback depicting the accumulated number of correct responses. Additionally, they were allowed to rest for a short period. Overall, participants were presented with approximately equal numbers of positive and negative feedback stimuli. At the end of the experiment, participants were told that they had performed very well – regardless of their points won – and all of them received a fixed monetary bonus. Subsequently, they were debriefed that no button press contingencies had existed throughout the experiment.

### Electroencephalographic Recording

The electroencephalogram (EEG) was recorded from 61 Ag/AgCl ring electrodes, arranged equidistantly in an electrode cap (EASYCAP GmbH, Herrsching, Germany; model M10). A balanced sterno-clavicular reference was used [61]. For off-line eye-movement correction, vertical and horizontal electrooculogram (EOG) was recorded bipolarily with electrodes placed on the outer canthi, and 1 cm above and below the left eye. Two pre-experimental eye-movement calibration trials were performed to calculate subject- and channel-specific weighted parameters for correction [62]. Electrode impedances were kept below 2 kΩ using a skin scratching procedure prior to EEG recording (see [63]). Signals were amplified using an AC amplifier set-up with a time-constant of 10 sec (Ing. Kurt Zickler GmbH, Pfaffstätten, Austria). All signals were recorded within a frequency range of 0.016 to 125 Hz and sampled at 250 Hz for digital storage.

Off-line and prior to analysis, the weighted EOG signals were subtracted from each EEG channel. So were individual blink coefficients, these were calculated using a template matching procedure (see [64]). EEGLAB 6.03b [65] was used for further analyses, e.g. low-pass filtering (cut-off frequency 30 Hz, roll-off 6dB per octave). Data segments of positive and negative facial feedback presentation were extracted; they started 100 ms before the respective stimulus onset and lasted for 700 ms each; the mean of the first 100 ms was used as baseline interval. Trials including gross muscular or movement artifacts were rejected via visual inspection before extended infomax independent component analysis (ICA) [66], [67] was performed to remove residual ocular artifacts, as described in [68]. Subsequently, a semi-automatic artifact removal procedure was applied to eliminate trials with voltage values exceeding +/−75 μV in any channel.

### Behavioral Data Analysis

Reaction times were defined as the interval from question mark onset to button press leading either to positive or negative feedback. Trials with reaction times faster than 100 ms were discarded from further analysis. Subsequently, reaction times were logarithmized by a natural logarithm function to achieve a more Gaussian distribution. The transformed reaction times were subjected to a mixed 2×3 repeated-measures analysis of variance (ANOVA) with the between-subject factor *group* (high-trait vs. low-trait) and the within-subject factor *cue* (100%, 75%, 0%). Furthermore, button choice behavior was assessed via calculating the number of rewarded choices throughout the experiment. An independent-samples t-test was used to test for group differences of button choice behavior.

### EEG Data Analysis

For each participant, artifact-free data segments of the feedback presentation were averaged per participant separately for positive facial stimuli (condition *pos*) and negative facial stimuli (condition *neg*). To assess the P1 component, mean amplitudes during 88–120 ms after facial stimulus onset were calculated at electrode site Oz for the conditions *pos* and *neg* first. Moreover, mean amplitudes at electrodes R26 and R27 (right hemisphere, corresponding to electrode locations in between T6/P6/P8 of the 10–20 system) were averaged together in the time interval of 88–120 ms after feedback onset for both conditions *pos* and *neg*, as were the mean amplitudes at L23 and 24 (left hemisphere, corresponding to electrode locations in between T5/P5/P7). To assess the N170 component, mean amplitudes at the same two electrode pairs (R26/R27 and L23/L24) were averaged together for the conditions *pos* and *neg* in the time interval of 160–180 ms after facial stimulus onset. Electrode locations for both P1 and N170 analysis were chosen based on recent literature [69], [70] and visual inspection of the data at hand. P1 latency was assessed from face onset to the corresponding positive maximum in the respective time window. Additionally, P300 mean amplitudes were assessed at electrode location Pz within the time range 300–600 ms after feedback onset.

To investigate early processing differences between the two groups, mixed 2×2×3 and 2×2×2 repeated-measure ANOVAs were conducted for P1 and N170 amplitude values, respectively. *Group* (high-trait, low-trait) served as between-subject factor, *valence* (pos, neg) and *electrode location* (right, middle, left for P1; right, left for N170) served as within-subject factors. For P300 amplitudes, *group* and *valence* served as factors for the ANOVA model. Note that feedback expectancy (i.e., expected vs. unexpected feedback) had no impact on these early ERPs when added as additional within-subject factor to the ANOVA models (all *F*'s<1), thus expectancy was not considered during analysis. Significant interaction effects were explored with Tukey HSD post-hoc tests. Furthermore, Pearson’s correlations were calculated between the mean amplitude values of each condition and electrode location and the individual T-scores of the AS-scale. The level of significance was set at *p*<.05 for all tests. Partial eta-squared (*η_p_^2^*) is reported to demonstrate effect sizes of the ANOVA models [71].
